# Refinement of breast cancer molecular classification by miRNA expression profiles

**DOI:** 10.1186/s12864-019-5887-7

**Published:** 2019-06-17

**Authors:** Rolf Søkilde, Helena Persson, Anna Ehinger, Anna Chiara Pirona, Mårten Fernö, Cecilia Hegardt, Christer Larsson, Niklas Loman, Martin Malmberg, Lisa Rydén, Lao Saal, Åke Borg, Johan Vallon-Christerson, Carlos Rovira

**Affiliations:** 1 Division of Oncology, Department of Clinical Sciences Lund, Lund University, Medicon Village, Scheelevägen 2, 223 81 Lund, Sweden; 2 Clinical Pathology, Laboratory Medicine, Skåne University Hospital, Lund, Sweden; 3 Division of Translational Cancer Research, Lund University, Lund, Sweden; 4 Department of Surgery, Skåne University Hospital, Lund, Sweden; 5 Division of Oncology, Skåne University Hospital, Lund, Sweden; 6 BioCARE, Strategic Cancer Research Program, Lund, Sweden; 7 German Cancer Research Center DKFZ, Division of Functional Genome Analysis, Heidelberg, Germany

**Keywords:** microRNA, Mir-4728, miR-99a/let-7c/miR-125b, LINC00478, Non-coding RNA, Differential expression, Breast cancer, Molecular subtypes

## Abstract

**Background:**

Accurate classification of breast cancer using gene expression profiles has contributed to a better understanding of the biological mechanisms behind the disease and has paved the way for better prognostication and treatment prediction.

**Results:**

We found that miRNA profiles largely recapitulate intrinsic subtypes. In the case of HER2-enriched tumors a small set of miRNAs including the HER2-encoded mir-4728 identifies the group with very high specificity. We also identified differential expression of the miR-99a/let-7c/miR-125b miRNA cluster as a marker for separation of the Luminal A and B subtypes. High expression of this miRNA cluster is linked to better overall survival among patients with Luminal A tumors. Correlation between the miRNA cluster and their precursor LINC00478 is highly significant suggesting that its expression could help improve the accuracy of present day’s signatures.

**Conclusions:**

We show here that miRNA expression can be translated into mRNA profiles and that the inclusion of miRNA information facilitates the molecular diagnosis of specific subtypes, in particular the clinically relevant sub-classification of luminal tumors.

**Electronic supplementary material:**

The online version of this article (10.1186/s12864-019-5887-7) contains supplementary material, which is available to authorized users.

## Background

Breast cancer is a heterogeneous disease and accurate classification of tumors into clinically relevant subgroups is critical for prognostication and treatment selection. The clinical management of breast cancer relies on parameters such as age, tumor size, and lymph node status, as well as on histopathological biomarkers including histological grade, expression of the hormone receptors estrogen receptor α (ER) and progesterone receptor (PR), and the presence or absence of amplification and concomitant overexpression of the human epidermal growth factor receptor 2 (HER2/ERBB2/neu, hereafter called HER2). But these biomarkers are still insufficient for accurate classification of patients into groups with high or low risk of recurrence and for identification of subgroups resistant to therapies. Technical advances during the last decades allowed for molecular stratification based on global gene expression. Messenger RNA (mRNA) expression profiles determined using microarrays demonstrated that breast tumors display unique intrinsic fingerprints that can be used to group tumors into intrinsic molecular subtypes [1–3]. This information has greatly improved our understanding of the heterogeneity of breast cancer and the different biological programs followed by the disease. Breast cancer can thus broadly be classified into five different molecular subtypes: Luminal A, Luminal B, HER2-enriched, Basal-like and a Normal breast-like group. Later, a subtype named Claudin-low has also been described [4]. The biology of these intrinsic subtypes reflects differences in incidence, response to treatment and survival and therefore specific genes can be tested as markers for each subtype to direct treatment options. Although great advances have been done in this field, there is still a need for new markers to refine classification, especially for certain subtypes. Most luminal tumors are ER-positive and HER2-negative (ER+/HER2-) but a subgroup of luminal B tumors is HER2+. Luminal A tumors are associated with a favorable clinical outcome, while luminal B tumors are clinically more aggressive with a higher rate of recurrence and lower survival rates. Still, luminal tumors are biologically heterogeneous and there are no clear-cut differences between the two major luminal subtypes. Luminal B tumors are more aggressive and higher expression of proliferation-related genes has been used as a positive marker for this disease type. However, no specific biomarkers have yet been proposed to discriminate tumors of luminal A type from luminal B and the group is therefore, on this point, largely defined by the absence of expression of proliferative genes. Comparisons of different gene signatures in breast cancer have been reported since long [5, 6]] and even for commercial tests agreement in classification has been suggested to be moderate in many instances [7] [8].

In this study, we wanted to explore whether miRNA expression could be informative for refinement of molecular subtypes. Expression profiles of miRNAs have previously been tested to help improving breast cancer subtyping, alone or integrated with mRNA profiles. In some instances they were suggested to be more informative than protein-coding RNA [9]. Iorio et al. showed that miRNA expression profiles produced by microarrays could clearly separate normal from cancer tissues [10]. Using bead-based flow cytometry, Blenkiron et al. [11] found that miRNA expression could accurately classify Basal versus luminal tumor subtypes. Later, Dvinge et al. [12] reported one of the most comprehensive studies of miRNA expression in breast cancer, including 1302 tumor samples from the Molecular Taxonomy of Breast Cancer International Consortium (METABRIC) study [13]. This project used microarrays for miRNA detection and created a resource that promoted a large number of follow-up studies. In recent years, next-generation sequencing of small RNAs has substituted all previously existing techniques for miRNA expression analysis. Next-generation sequencing offers the advantages of higher sensitivity and a less biased view of the small RNA transcriptome since it is not limited to a set of predetermined miRNA genes. Importantly, the introduction of next-generation sequencing has led to a dramatic increase in the number of known human miRNAs, most of which were not present on the microarrays that were used in earlier studies of miRNA expression in cancer. In the present study we have therefore applied next-generation sequencing to study the small RNA expression profiles of a collection of 186 tumors from the Sweden Cancerome Analysis Network – Breast (SCAN-B) initiative [14] to identify miRNA profiles that could improve classification of breast cancer subtypes.

## Results

### MicroRNA expression in the cohort

We performed small RNA sequencing for a total of 186 tumor samples from the SCAN-B initiative, including samples representing all intrinsic subtypes except for Normal-like (Table 1). Illumina sequencing produced an average of 4.9 million aligned reads per sample, with 77% of all aligned reads between 19 and 25 nt in length and 73% corresponding to miRNAs (Additional file 2: Figure S1 and S2). The mean number of expressed miRNAs per sample was 684 and the most highly expressed miRNA was miR-21-5p, overexpressed in most cancers and one of the best-characterized oncogenic miRNAs in breast tumors. Mean expression profile was calculated and plotted to compare the complexity of miRNA expression profiles among individual samples (Additional file 2: Figure S3). The expression profiles of the cohort are highly uniform with a comparable number of expressed miRNAs within different expression intervals.

**Table 1:** Characteristics of the study cohort. Number of patients classified according to PAM50 subtypes (Basal, HER2, Luminal A or Luminal B), estrogen receptor, progesterone receptor and HER2 receptor status

				n
Basal	ER-	HER2-	PR-	45
Basal	ER-	HER2+	PR-	4
Her2	ER-	HER2-	PR-	1
Her2	ER-	HER2+	PR-	29
Her2	ER+	HER2-	PR-	1
Her2	ER+	HER2-	PR+	3
Her2	ER+	HER2+	PR-	5
Her2	ER+	HER2+	PR+	3
LumA	ER-	HER2-	PR-	1
LumA	ER+	HER2-	PR+	30
LumA	ER+	HER2+	PR+	9
LumB	ER-	HER2-	PR-	1
LumB	ER+	HER2-	PR+	36
LumB	ER+	HER2+	PR-	4
LumB	ER+	HER2+	PR+	12

### Unsupervised clustering analysis identifies functionally distinct tumor subtypes

**Fig. 1:**
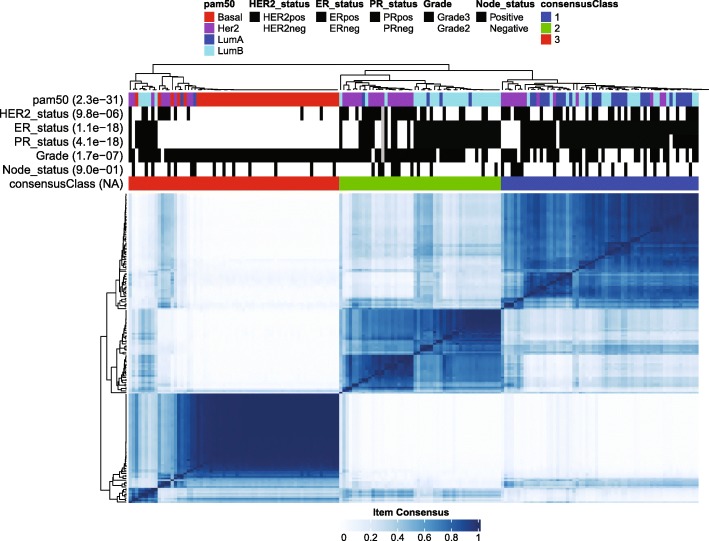
Consensus clustering identified three main tumors clusters with distinct sample composition. Enrichment of PAM50 subtypes and clinical parameters within cluster was evaluated using the χ^2^ test and p-values are given in parenthesis

A ConsensusCluster analysis based on miRNA expression revealed the presence of three major clusters of tumor samples (Fig. 1 and Additional file 2: Figure S4). We tested for significant association between these groups and commonly used clinical markers and found that PAM50 subtype had the most significant enrichment. Also ER, PR and HER2 status are highly significantly associated with sample clustering. Consensus cluster 1 is enriched for Luminal A samples and few of tumors with this subtype are located in the other two clusters. Luminal B samples are concentrated in consensus cluster 2, but are also found in cluster 1 along with the Luminal A subtype. HER2-enriched tumors form small sub-clusters within consensus cluster 1 but principally concentrate in cluster 2. Strikingly, all samples of the Basal subtype cluster together in consensus cluster 3 (Fig. 1). A small number of samples from other PAM50 subtypes also cluster together with the Basal-like tumors in consensus cluster 3, but most of these have lower item consensus scores, indicating a less confident cluster membership. Genomic Grade Index (GGI) has been shown to be associated with PAM50 classes and is a measurement strongly associated with cellular proliferation [15]. Almost all GGI high samples are distributed among consensus clusters 2 (mainly HER2-enriched and Luminal B/ER+) and 3 (mainly Basal-like tumors), while GGI low samples are located in consensus cluster 1, coincident with the lower proliferative activity of Luminal A tumors (data not shown). These results show that our miRNA expression data reflect the functional differences behind subtypes and clinical characteristics of these breast tumors.

### MicroRNA expression clusters associated with PAM50 subtypes

Next, we wanted to identify miRNAs that were associated with different tumor subtypes. Two samples were excluded from this analysis since they displayed low correlation to subtype centroids and was not assigned to a PAM50 class, the other samples were distributed as follows: Basal-like = 49, HER2-enriched = 42, Luminal A = 40, and Luminal B = 53 (Table 2). Differential miRNA expression analysis was performed using edgeR [16], both for comparisons between respective subtype and the rest, as well as for all possible pairwise combinations of subtypes. These results are summarized in Additional file 1: Table S1.

**Table 2:** Characteristics of the study cohort. Number of patients according to age and TNM staging. T1-T4 increasing tumor size and extent. T1 = smallest tumor. N = lymph node status, N0 = no tumor in the lymph nodes**,** N1-N3 = increasing number of nodes affected. M0-M1 = No distant mestastases (0) or distant metastases have been found (1)

pam50	mean age	T1	T2	T3	N0	N1	N2	N3	NX	M0	M1	MX
Basal	65	23	35	1	50	9	0	0	0	50	0	9
Her2	66	22	23	0	25	15	3	1	1	41	1	3
LumA	63	21	18	1	32	6	2	0	0	33	0	7
LumB	65	25	30	2	47	9	1	0	0	53	1	3

**Fig. 2:**
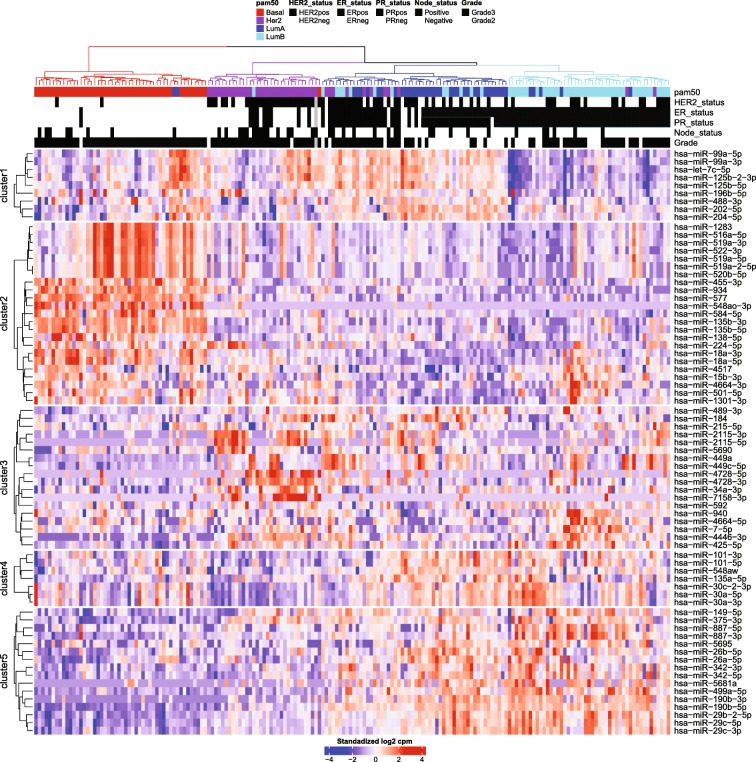
Sample clustering and heatmap for the expression of a set of 73 unique miRNAs collected from the 15 most significantly differentially expressed miRNAs from each comparison between tumor subtypes. Samples are colored by subtype according to the PAM50 classification. Red = Basal-like, purple = HER2-enriched, blue = Luminal A, and cyan = Luminal B. The miRNA expression values were standardized by row mean centering and dividing by row standard deviation in R before distance calculation and clustering

Previously published work failed to find a significant correlation between miRNA profiles and the intrinsic subtypes. For example, in The Cancer Genome Atlas (TCGA) basal tumors could be classified according to miRNA expression, while the Luminal A, Luminal B and HER2-enriched tumors formed a mixed group with no or low correlation with the PAM50 subtypes [17]. Here, we used the entire list of miRNA genes registered in miRbase 22 [18] to identify signatures of differentially expressed miRNAs associated with the different subtypes. We identified a total of 655 unique microRNAs that were significantly differentially expressed between the intrinsic subtypes. We focused our analysis on the most significant miRNAs and identified 73 unique miRNAs that were clearly differentially expressed between intrinsic subtypes. When this set of selected miRNA was used for supervised clustering the tumors formed four well-defined clusters and the genes formed five separate miRNA clusters (Fig. 2).

As expected, clusters with luminal tumors are associated with ER-positivity, while clusters with the Basal-like and most HER2-enriched tumors are ER-negative (Fig. 2). ER+ tumors are mainly characterized by high expression of mir-26, mir-5681a, mir-5695, mir-887, mir-149, mir-375, mir-342, mir-190b, mir-29c, mir-29b and mir-499a (miRNA cluster 1). Some of these genes has already been observed to be upregulated in breast cancer and seems to be a characteristic of ER+ cancers [19]. Concomitant with the upregulation of these genes, downregulation of mir-455-3p, mir-934, mir-135b and mir-577 can be observed among ER+ samples (miRNA cluster 2). These miRNAs are in turn upregulated among ER- tumors. ER- tumors are also characterized by overexpression of mir-18a (cluster 3). Mir-18a is encoded by the pri-mir-17~92 locus and the mature product, miR-18a-5p, represses expression of ERα directly by binding to its mRNA [20]. The ER+ miRNA expression signature is the driving force that segregates luminal tumors into a separate branch.

Basal-like tumors form a relatively homogenous group characterized by high expression of mir-548ao, mir-584, mir-138, mir-135b, mir-455, mir-577, and mir-934 (miRNA cluster 2). The basal-like cluster can in turn be subdivided by the expression of mir-516a, mir-519a, mir-520b, mir-522, and mir-1283, which are all part of a large primate-specific miRNA cluster on chromosome 19 referred to as C19MC [21]. Further subdivision of the Basal-like tumors depends on the expression of the mir-99a/let-7c/mir-125b-2 miRNA cluster. Basal-like tumors are mostly triple negative (ER−/PR−/HER2-) and also have low expression of the miRNAs that are part of the ER+ signature, such as mir-29c, mir-190b, and mir-499a. Interestingly, mir-548ao is exclusively expressed in Basal-like tumors. This miRNA belongs to a large primate/human-specific family [22] of miRNA genes derived from the short miniature inverted-repeat transposable elements Made1.

As previously reported [17], there is only partial overlap between the PAM50 HER2-enriched subtype and clinically determined HER2+ tumors. Most of the tumors classified as HER2-enriched using the PAM50 signatures cluster together and are mainly defined by high expression of three miRNA genes: mir-34a, mir-2115, mir-4728, and mir-7158. MicroRNA-4728 is encoded inside the *HER2* oncogene itself and has been shown to be upregulated together with its host gene [23, 24]. Interestingly, mir-7158 is strongly associated with the HER2-enriched subtype and mainly ER- tumors, but not with the histologically determined HER2+ samples. In fact, just as for mir-548ao in triple negative tumors, mir-7158 is hardly expressed in any other tumor type, indicating a strong functional association with the HER2-enriched profile and not necessary with the overexpression of the *HER2* oncogene itself. This miRNA is embedded inside the non-coding RNA gene LINC01105, a non-coding RNA almost exclusively expressed in brain and probably regulate the expression of HIF-1α [25, 26]. Expression of mir-2115 is also associated with HER2-enriched tumors but displays a gradient of expression with samples having a luminal profile at the higher end of expression. Contrary to mir-4728 [23, 24, 27–29], mir-2115 and mir-7158 have been poorly characterized. We therefore investigated their processing pattern using public data and found that their reads originate from true miRNA precursors [30](Additional file 2: Figure S5). HER2+ tumors outside the HER2-enriched cluster always have high expression of mir-4728. A group of HER2+ tumors segregate together with Luminal B tumors and have a distinct miRNA profile (miRNA cluster 3). All of these tumors are ER+.

We used the microRNAs identified in our dataset to cluster TCGA breast cancer samples as well as the normal samples available. We found that mature microRNAs from mir-4728, mir-2115 and mir-7158, were also enriched in the pam50 HER2 classified samples. The basal samples were also clustered strongly based on the selected microRNAs. The luminal A/B samples cluster also well, but with multiple distinct groups of the Luminal A samples (Additional file 2: Figure S6).

Tumors belonging to the Luminal A and B subtypes have similar expression profiles, characterized by high expression of the previously mentioned set of ER-associated miRNAs (miRNAs in cluster 1). But strikingly, the miRNAs grouped in expression cluster 5 are differentially expressed between Luminal A and B tumors. These miRNAs could be useful for the separation of these two breast cancer subtypes. Due to the clinical importance of this distinction, we performed a focused analysis for these two subtypes.

### Upregulation of miR-99a/let-7c/miR-125b is a characteristic of luminal a tumors

**Fig. 3:**
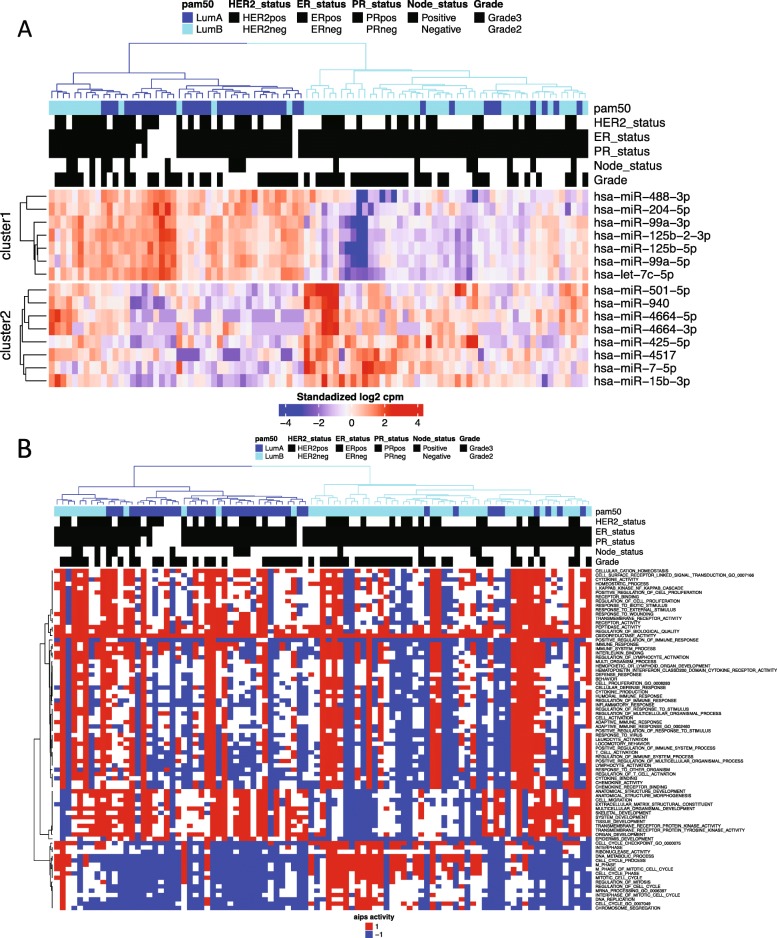
**a** Sample clustering and heatmap for the 15 most significant miRNAs differentially expressed between luminal A (blue) and luminal B (cyan) samples. **b** Corresponding gene signature activation using absolute inference of patient signatures (AIPS) model. The AIPS model uses gene expression signatures in the mRNA dataset to identify active and inactive biological processes. The AIPS algorithm assigns a random or independent value to samples, which do not show a clear active nor in-active process. Red = active, blue = inactive, white = Random/Independent

Molecular classification of luminal ER+ tumors based on mRNA expression profiles is not clear-cut; Luminal B tumors typically have higher proliferation and PAM50 classification is therefore mainly driven by the expression of proliferation-related genes. Luminal A tumors are characterized by high expression of ER and PR, but low expression of proliferation markers. Using molecular data, the criterion is a relative comparison. The distinction between luminal samples would benefit from Luminal A associated and up-regulated genes that could provide positive markers for the subtype, complementing the characteristic of low expression of proliferation markers. Given the clustering of tumors by subtype shown in Fig. 2, we wanted to analyze if miRNA expression profiling could identify such markers. As shown in Fig. 3a, the miRNA cluster mir-99a/let-7c/mir-125b-2 is upregulated in Luminal A tumors compared with Luminal B. This miRNA cluster is located on chromosome 21 and the encoded miRNAs are candidate tumor-suppressors implicated in the regulation of inflammation and stem-like properties [31–33]. Since Luminal B tumors are associated with high expression of proliferation-associated genes we wanted to test if this was also the case for the classification of luminal tumors by miRNA profiles. A χ2 test for pathways registered in the Molecular Signatures Database [34] confirmed that the most significant pathways associated with the separation of these tumors according to their miRNA expression are related to proliferation (Fig. 3b).

**Fig. 4:**
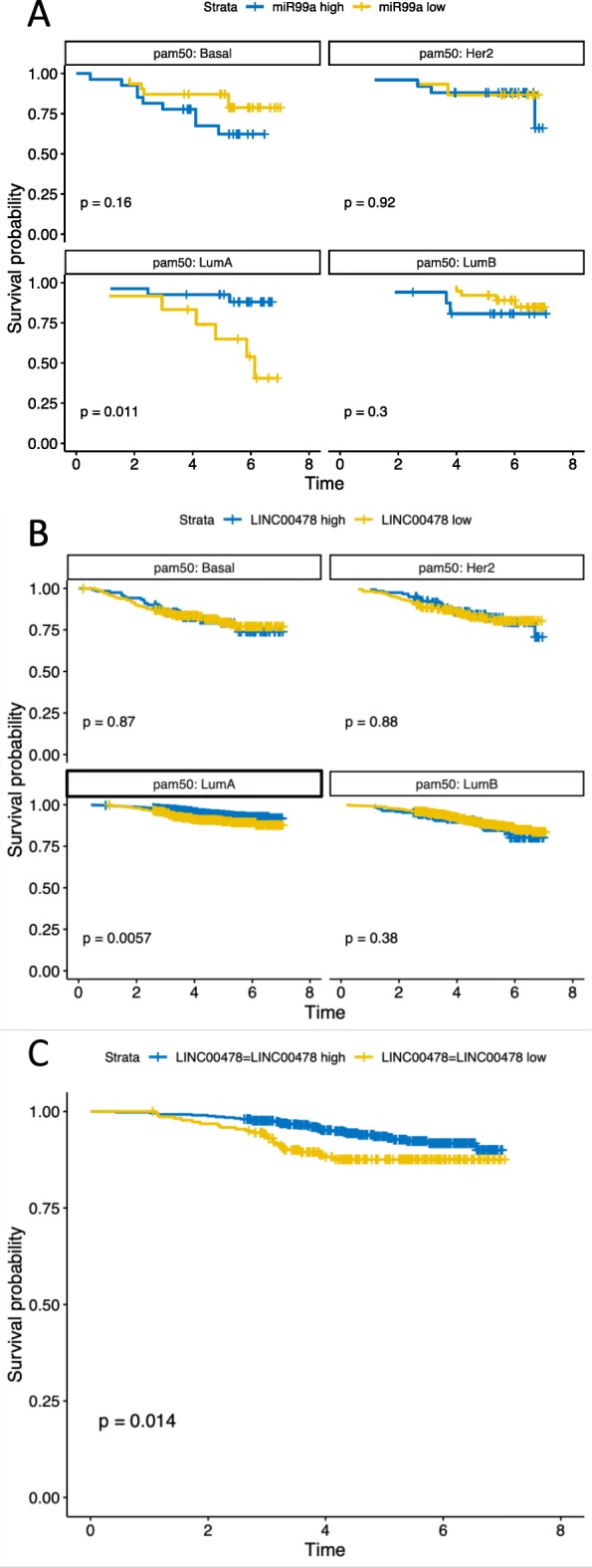
**a** Survival analysis based on the mean expression of the microRNAs in the miR-99a/let-7c/miR-125b miRNA cluster. We used the median to split the patients into miR99a high (higher than median expression) and miR-99a low (lower than median expression). These groups were used in a survival analysis with R survival package, stratified based on the pam50 group and plotted using the survminer package in R. **b** We used the median to split the patients into LINC00478 high (higher than median expression) and LINC00478 low (lower than median expression). These groups were used in a survival analysis with R survival package, stratified based on the pam50 group and plotted using the survminer package in R We found a significant benefit for Luminal A breast cancer patients of having higher than median LINC00478 expression. **c** A focused analysis was done on the Luminal A samples, with ER+, HER2-, node negative, endocrine treatment, no chemotherapy and no anti-HER2 treatment. This subgroup represents 725 patients

Next, we performed a survival analysis within each PAM50 Luminal subtype stratifying samples according to high or low mir-99a/let-7c/mir-125b-2. The survival curves are shown in Fig. 4a. Luminal A patients with low expression of mir-99a/let-7c/mir-125b-2 have significantly lower overall survival. There is no significant effect among patients with Luminal B type cancer. Together, these results suggest that expression of mir-99a/let-7c/mir-125b-2 is a candidate biomarker for separating Luminal A and B tumors, and that this miRNA cluster is a prognostic marker within the Luminal A subtype.

The mir-99a~let-7c~mir-125b-2 cluster is expressed from the primary miRNA cluster MIR99AHG, encoded in a non-coding RNA host gene called LINC00478. We find that the averaged expression of miRNAs in the cluster is correlated with expression of MIR99AHG /LINC00478 measured in our mRNA expression analysis. This correlation is highly significant (*p*-value < 1*10 ^-15) with a slope of 0.40, indicating a better dynamic range in the detection of the microRNAs (Additional file 2: Figure S7). We therefore used LINC00478 expression in the survival analysis of all patients in the mRNA cohort. The median LINC00478 expression was used as cutoff for the survival analysis. We found the same significant result for the Luminal A breast cancer patients, as for the miRNA cohort (Fig. 4b). The stratification remained significant for luminal A cases when restricted to node negative patients who received endocrine treatment but no chemotherapy or anti-HER2 treatment (Fig. 4c). Of note, we also found that only patients who did not receive radiotherapy had a survival benefit of having higher levels of mir-99a cluster (Additional file 2: Figure S8).

## Discussion

The discovery that breast cancer can be classified into distinct intrinsic molecular subtypes has been important both for research and in the clinic. Many different technologies for gene expression analysis have been tested for identification of tumor subtypes. Several prognostic gene signatures have been reported and some are commercially available. However, there are certain discrepancies regarding their accuracy and the reproducibility in part due to differences of cohorts but also experimental platforms used [35]. In this study we have revisited the classification of breast tumors into intrinsic subtypes using miRNA expression data from high-throughput sequencing and comprehensive miRNA annotation. Early reports on miRNA signatures in breast cancer were limited by the number of human miRNAs that had been identified at the time, and by the resulting probe sets printed on microarrays or primers designed for quantitative reverse transcriptase PCR (qRT-PCR). Moreover, much of the published miRNA research has focused on broadly conserved miRNAs while evolutionarily younger, species-specific miRNAs have been largely disregarded. In humans only a few hundred miRNAs are shared with the closest relatives outside the great apes and the young, non-conserved miRNAs by far outnumber conserved miRNA genes. The central repository of annotated miRNAs in all species, miRBase, has a high-confidence dataset that is limited to miRNAs which meet a number of criteria regarding minimal expression levels and structure. Not all reported human miRNA genes fulfill these criteria and these are typically the non-conserved miRNAs that are lowly expressed and sometimes restricted to specific tissues or developmental stages. However, cancer is a disease of the genome and genomic aberrations that can deregulate miRNA expression are common [36, 37]. Here we show that HER2-enriched tumors are characterized by high and specific expression of three normally lowly expressed, non-conserved miRNAs: mir-2115, and mir-7158 and mir-4728. We have previously reported that mir-4728 is encoded within the HER2 oncogene and that it is co-amplified with it in HER2+ breast cancer [23, 24]. Since this miRNA is poorly conserved and very lowly expressed in most normal tissues, it is often excluded from genome-wide studies. However, both we and others have shown that mir-4728 is not only co-expressed with HER2 but also act as an oncogene in HER2+ tumors [23, 27, 38]. This is a reminder that miRNA studies in cancer should consider all human miRNA genes, not just a high-confidence set, since also non-conserved miRNAs can serve as biomarkers and have equally important regulatory functions in disease. Moreover, previous work has suggested that miRNA/host co-transcription in breast cancer is limited to a small portion of the known intron-encoded miRNAs [12]. Therefore, since clinically important signatures in general are based on mRNA profiles, the absence of co-expression would prevent the direct translation of miRNA expression into clinically relevant signatures. Yet, we show here that just as mir-4728 is a perfect surrogate marker for HER2 expression [39], the miR-99a/let-7c/miR-125b cluster is co-transcribed with the LINC00478 gene which may serve as an expression surrogate in luminal samples. This latter observation may also have clinical implications.

Tumors of the Luminal A and Luminal B subtypes express high levels of ER. Patients with these subtypes consequently receive endocrine therapy and optionally in combination with adjuvant chemotherapy. Accurate classification of ER+/HER2- Luminal tumors into low- and high-risk groups is an important clinical issue since it could help guide treatment decisions. The positive effects of anti-hormonal treatment are well established for ER+ cancer, whereas chemotherapy, that is associated with worse side effects and higher healthcare costs as well as socioeconomic cost, should only be used in patients that will benefit from the treatment. Luminal A tumors have a low proliferation rate, while luminal B neoplasms are more aggressive and associated with a significantly worse prognosis. A better classification of luminal ER+/HER2- tumors may therefore aid in minimizing overtreatment by identifying those breast cancers that can be successfully treated with endocrine therapy alone. The major discriminator between the Luminal A and B subtypes is the expression of proliferation-associated genes but their expression is not simply bimodal. They essentially display a continuous gradient of expression values making it difficult to establish a clear cut-off between the two subtypes. For lack of positively expressed biomarkers, Luminal A samples are identified by the absence of a proliferative gene signature [40]. Ki67 has been proposed as a prognostic proliferation marker for luminal breast cancer, but its clinical value remains uncertain due to problems with the interlaboratory variability of immunohistochemistry and scoring, as well as, since it too is a continuous variable, the lack of an clear cut-off between high and low proliferation [41, 42]. Unfortunately, Ki67 staining was only available for a subset of the samples included in this cohort, but the miRNA profiles of luminal A tumors were associated with lower GGI indexes, which is also a measurement strongly associated with cellular proliferation. Here we show that Luminal A miRNA expression signatures were characterized by higher expression of miRNAs that act as inhibitors of proliferation and which are known tumor suppressors. We observed that luminal A tumors have higher expression of the mir-99a/let-7c/mir-125b-2 miRNA cluster in comparison with luminal B samples. Patients with high mir-99a expression have better survival than those expression low levels. This is consistent with earlier observations by Bailey et al. [43]. The let-7 miRNA family has been shown to be involved in the downregulation of proteins in multiple pro-proliferative signaling pathways, including JAK, STAT3, c-Myc and RAS. Also mir-99a is a known tumor suppressor that controls cell proliferation by inhibition of AKT/mTOR signaling. The third miRNA, mir-125b may have oncogenic or tumor-suppressive effects depending on the cell context, but has been shown to downregulate expression of pro-proliferative genes such as *ETS1* [44]. In addition to these observations, correlation between the miRNA cluster and their precursor LINC00478 is highly significant, suggesting that its expression could help improve the accuracy of present day’s signatures to help discriminating luminal samples. Taken together, the findings propose that Luminal A patients with high LINC00478 expression might not benefit from chemotherapy treatment and therefore could be spared this added treatment.

In summary, a comprehensive miRNA expression profiling using the complete set of currently annotated human miRNAs from miRBase can help to refine subtype classification in breast cancer. These results also indicate that miRNA signatures can be directly translated to mRNA surrogates, opening new opportunities for identifying clinically applicable markers for improved stratification and diagnostics of breast tumors.

## Conclusions

Breast cancer is a heterogeneous disease. For appropriate clinical management and to help develop better therapies, classification of different subtypes should be based on underlying biological properties. Molecular stratification based on mRNA expression of an intrinsic gene list uncovered five subtypes. We used microRNA expression profiles to refine these major subtypes. Here we show that miRNA expression can facilitate the molecular diagnosis of specific subtypes, in particular the clinically relevant sub-classification of luminal tumors.

## Methods

### Collection of specimens

One hundred and eighty six samples were selected from a larger cohort with the aim of creating four relatively equally sized groups with respect to ER- and HER2-status. To minimize variation between groups, tumors with histological grade 1 and tumors of the normal-like subtype were excluded. The final set contained 54 ER+/HER2+, 53 ER+/HER2-, 24 ER−/HER2+, and 55 ER−/HER2- tumors (Table 1).

### PAM50 classification

The PAM50 subtyping was performed by nearest-centroid method using the centroids from Parker et al. [45] and using mRNA gene expression data from RNAseq. To avoid cohort dependency when assessing nearest-centroid and assigning PAM50 subtype, the gene expression for each sample was normalized back to the original training cohort by gene centering against a fixed reference set of samples selected to match the clinical characteristics of the cohort used by Parker et al. [45].

### Small RNA sequencing

Small RNA sequencing was done as described in Persson et al. [46]. Briefly, total RNA was extracted from aliquots of the same tumor homogenates used for column-based extraction and mRNA-sequencing in [14] but using TRIzol LS (Thermo Fisher Scientific) according to the manufacturer’s instructions and concentrations were measured on a NanoDrop ND 1000 spectrophotometer (NanoDrop Tech). Small RNA sequencing libraries where prepared from 500 ng total RNA with custom adapters to incorporate dual indexes. Pooled libraries were sequenced on a NextSeq 500 with High Output v2 75 cycle-kits (Illumina).

Sequences were demultiplexed using Picard and aligned against the hg38 human genome assembly using Novoalign with settings -a TGGAATTCTCGGGTGCCAAGG -l 14 -h − 1 -1 -t 90 -g 50 -× 15 -o SAM -o FullNW -r All 51 -e 51. These alignment settings allow for mismatches, insertions or deletions of up to 3 nt to accommodate for e.g. non-templated nucleotide additions. One sample with fewer than 500,000 reads was excluded from further analysis. MicroRNA expression profiles were generated for miRBase version 22 [47] using software developed in-house. Briefly, the mapped coordinates of each read were compared to the annotated positions of mature miRNAs and counts were assigned to a given miRNA if the coordinates overlapped with a maximum total deviation of 4 nt, excluding mismatches at the read start and end. Multi-mapping reads were excluded unless all genomic matches mapped to annotated miRNAs with the same mature miRNA sequence. Annotation errors in miRBase release 22 where identical IDs were assigned to distinct primary miRNA loci for *mir-4477a*, *mir-4477b* and *mir-10,401* were corrected in our input annotation files.

### Data analysis

Counts per mature miRNA were normalized to counts per million reads with addition of a pseudo-count to avoid zero values before log_2_-transformation with the function cpm(x, normalized.lib.sizes = TRUE, log = TRUE, prior.count = 0.25) of the edgeR R package [48]. The number of expressed miRNAs was calculated for the expression interval from − 5 to 20 on the normalized log_2_-transformed scale. The ConsensusClusterPlus [49] R package was used with the following settings (maxK = 6, reps = 1000, pItem = 0.8, pFeature = 1, innerLinkage = “average”, finalLinkage = “average”, clusterAlg = “hc”, distance = “pearson”). The item consensus matrix of the consensus clustering was replotted with the ComplexHeatmap [50] R package with annotation and χ^2^ *p*-values for enrichment in the identified clusters. PAM50 subtyping was performed by nearest-centroid using centroids from Parker et al. [45]. Before assessing nearest-centroid, the expression data for each sample was adjusted by gene-centering against a fixed reference cohort selected to match the clinical characteristics of the original cohort used by Parker et al. The gene centering was done to account for differences in cohort composition that may otherwise result in biased subtype classifications [51]. The edgeR package was used to identify miRNAs differentially expressed between PAM50 subtypes. Two approaches were used to find significant miRNAs; one-group vs rest and pairwise contrasts between all groups. The ComplexHeatmap package was used to make heatmaps with the settings (clustering_distance_rows/columns = “Euclidean”, clustering_method_rows/columns = “ward.D2”) and the dendextend [52] R package was used to annotate and color the dendrogram. The number of clusters for the rows/genes/miRNAs was based on iterative plotting and evaluation.

### Survival analysis

Overall survival was used as endpoint in survival analysis. Stratification of patients into groups of either high or low expression was done by median expression value as cut-point. Stratification based on miRNA expression was done using average levels of the miR-99a~let-7c~miR-125b cluster. Stratification based on mRNA expression was done using expression level of LINC00478. A univariate survival analysis was performed using the survival package in R. The survival curves were plotted with the survminer package in R. Analysis was performed stratified based on PAM50 molecular subtype. In the focused analysis we selected patients with PAM50 molecular subtype LumA that were: ER+, HER2-, node negative, administered endocrine treatment but not treated with chemotherapy nor anti-HER2 treatment.

### TCGA analysis

For calculation of miRNA expression for TCGA data, miRNA isoform quantification files based on hg38 and miRBase 21 were downloaded for all available samples using the GDC command line client. All isoform coordinates were then matched to miRBase 22 coordinates allowing up to 4 bp deviation from the annotated mature sequence. Isoform read counts were then reassigned to each corresponding mature miRNA. Note that the TCGA isoform quantification files use a unique coordinate system (1-based, half-closed/half-open) which includes the start coordinate and excludes the end coordinate.

## Additional files

Additional file 1: **Table S1.** Summary of differentially expressed microRNAs. The selected cutoff for significance was FDR 0.01. (XLSX 2878 kb)

Additional file 2: **Figure S1.** Boxplot summarising sequencing statistics including total purity-filtered reads, uniquely aligned reads, multimapping reads, unaligned reads and reads that were removed due to a very short insert size (< 14 nt) across all libraries. **Figure S2.** Boxplot summary of the insert size on top for the aligned reads and summary of the composition based on RNA class. **Figure S3.** Cumulative counts of expressed miRNAs in the expression interval − 5 to 20 log_2_ counts per million reads (cpm) show that all sequenced libraries have a high and similar miRNA profile complexity. The intervals are spaced by 0.5 log_2_ cpm. The individual samples are plotted in grey and the mean sample is plotted in black. **Figure S4.** Plots from consensusclustering analysis. The number of k clusters (k = 3) was identified from the delta area plot. As observed the increase in consensus with the number of clusters. The increase in consensus is low for k = 4 and therefore k = 3 was used. **Figure S5.** Expression pileups for mir-2115 and mir-7158 from miRCarta. **Figure S6.** Clustering of TCGA breast cancer using the miRNAs identified in our analysis. **Figure S7.** Correlation of an average of the microRNAs in the MIR99AHG cluster (mir-99a, let-7c and mir-125b-2) and the LINC00478 (MIR99AHG) from the mRNA expression cohort. The values are mean centered to ease the comparison. The slope is 0.4 indicating a better dynamic range for the detection of the microRNAs. **Figure S8.** A focused analysis on the Luminal A samples with stratification on whether or not the patient has received radiotherapy. (DOCX 3368 kb)

## Data Availability

The data required to replicate the findings of this study are available from NCBI Gene Expression Omnibus (GEO) under accession number GSE131599 and from the corresponding author on request.

## Abbreviations

AIPS: Absolute inference of patient signatures
ER: Estrogen receptor
HER2: Human epidermal growth factor receptor 2
miRNA: microRNA
PR: Progesterone receptor
SCAN-B: Sweden Cancerome Analysis Network-Breast
