# Supplementary material for: Refinement of breast cancer molecular classification by miRNA expression profiles
Source: BMC Genomics. 2019 Jun 17;20:503. doi: 10.1186/s12864-019-5887-7 (PMC6580620; doi:10.1186/s12864-019-5887-7)
Supplement: Supplementary file 2 — Figure S1. Boxplot summarising sequencing statistics including total purity-filtered reads, uniquely aligned reads, multimapping reads, unaligned reads and reads that were removed due to a very short insert size (< 14 nt) across all libraries. Figure S2. Boxplot summary of the insert size on top for the aligned reads and summary of the composition based on RNA class. Figure S3. Cumulative counts of expressed miRNAs in the expression interval − 5 to 20 log2 counts per million reads (cpm) show that all sequenced libraries have a high and similar miRNA profile complexity. The intervals are spaced by 0.5 log2 cpm. The individual samples are plotted in grey and the mean sample is plotted in black. Figure S4. Plots from consensusclustering analysis. The number of k clusters (k = 3) was identified from the delta area plot. As observed the increase in consensus with the number of clusters. The increase in consensus is low for k = 4 and therefore k = 3 was used. Figure S5. Expression pileups for mir-2115 and mir-7158 from miRCarta. Figure S6. Clustering of TCGA breast cancer using the miRNAs identified in our analysis. Figure S7. Correlation of an average of the microRNAs in the MIR99AHG cluster (mir-99a, let-7c and mir-125b-2) and the LINC00478 (MIR99AHG) from the mRNA expression cohort. The values are mean centered to ease the comparison. The slope is 0.4 indicating a better dynamic range for the detection of the microRNAs. Figure S8. A focused analysis on the Luminal A samples with stratification on whether or not the patient has received radiotherapy. (DOCX 3368 kb) [file 12864_2019_5887_MOESM2_ESM.docx]

**Supporting Information**

**Refinement of breast cancer molecular classification by miRNA expression profiles**

Rolf Søkilde, Helena Persson, Anna Ehinger, Anna Chiara Pirona, Mårten Fernö, Cecilia Hegardt, Christer Larsson, Niklas Loman, Martin Malmberg, Lisa Rydén, Lao Saal, Åke Borg, Johan Vallon-Christerson and Carlos Rovira

**Additional file 1: Figure S1.** Boxplot summarising sequencing statistics including total purity-filtered reads, uniquely aligned reads, multimapping reads, unaligned reads and reads that were removed due to a very short insert size (< 14 nt) across all libraries.

**Additional file 1: Figure S2.** Boxplot summary of the insert size on top for the aligned reads and summary of the composition based on RNA class.

**Additional file 1: Figure S3.** Cumulative counts of expressed miRNAs in the expression interval -5 to 20 log_2_ counts per million reads (cpm) show that all sequenced libraries have a high and similar miRNA profile complexity. The intervals are spaced by 0.5 log_2_ cpm. The individual samples are plotted in grey and the mean sample is plotted in black.

**Additional file 1: Figure S4.** Plots from consensusclustering analysis. The number of k clusters (k = 3) was identified from the delta area plot. As observed the increase in consensus with the number of clusters. The increase in consensus is low for k =4 and therefore k = 3 was used.

mir-2115

<https://mircarta.cs.uni-saarland.de/detailed_expression/MI0010634/>


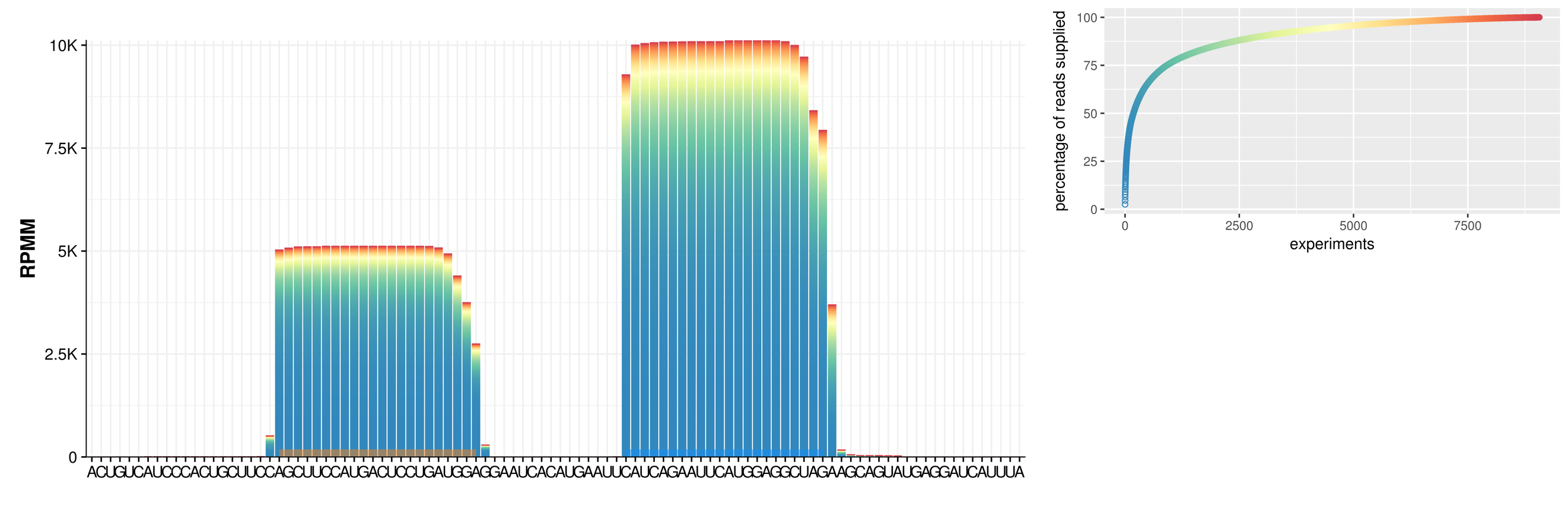


mir-7158

<https://mircarta.cs.uni-saarland.de/detailed_expression/MI0023618/>


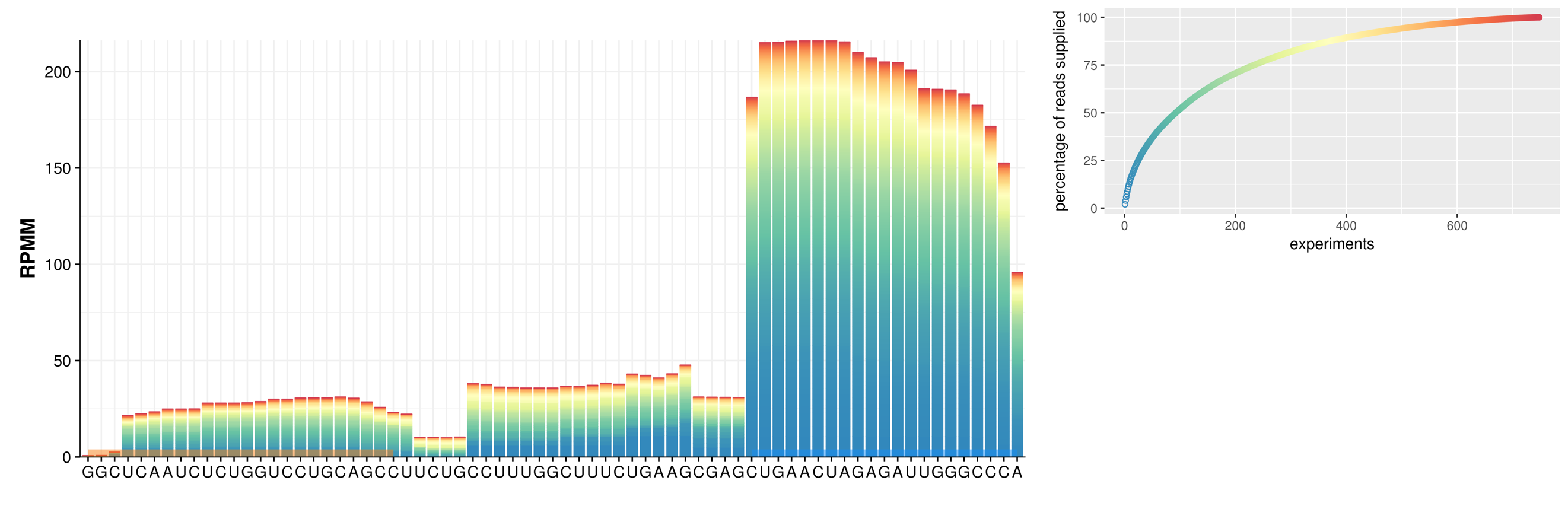


**Additional file 1: Figure S5.** Expression pileups for mir-2115 and mir-7158 from miRCarta.


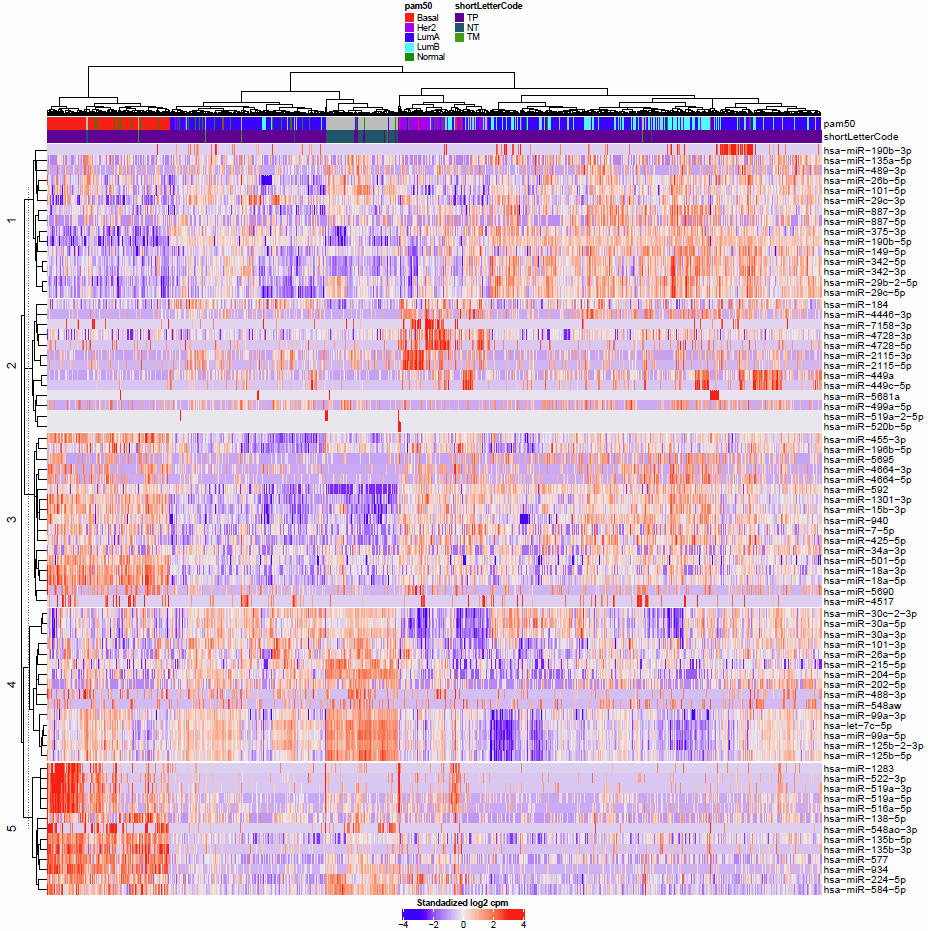


**Additional file 1: Figure S6.** Clustering of TCGA breast cancer using the miRNAs identified in our analysis.

**Additional file 1: Figure S7.** Correlation of an average of the microRNAs in the MIR99AHG cluster (mir-99a, let-7c and mir-125b-2) and the LINC00478 (MIR99AHG) from the mRNA expression cohort. The values are mean centered to ease the comparison. The slope is 0.4 indicating a better dynamic range for the detection of the microRNAs.

**Additional file 1: Figure S8.** A focused analysis on the Luminal A samples with stratification on whether or not the patient has received radiotherapy.
